# The dark and bright side of laissez-faire leadership: Does subordinates’ goal orientation make a difference?

**DOI:** 10.3389/fpsyg.2023.1077357

**Published:** 2023-03-15

**Authors:** Jiaojiao Zhang, Yao Wang, Feng Gao

**Affiliations:** ^1^School of Business, Renmin University of China, Beijing, China; ^2^School of Business, China University of Political Science and Law, Beijing, China

**Keywords:** laissez-faire leadership, goal orientation, challenge appraisal, hindrance appraisal, job performance

## Abstract

Laissez-faire leadership is universally considered to be the most ineffective leadership style. However, a few recent studies revealed that laissez-faire leadership may have modest or even significant positive influence on subordinates’ work outcomes. To explain the inconsistent findings of laissez-faire leadership studies, the current study draws on stress theory and achievement goal theory to examine the boundary conditions and mechanisms underlying the impact of laissez-faire leadership on subordinates, cognitive appraisal and subsequent performance. Results from an experience sampling study of 68 supervisor-subordinate dyads that completed daily surveys over 10 consecutive work days indicated that: (1) when subordinates’ learning goal orientation is high, the relationship between laissez-faire leadership and hindrance appraisal will be positive; the indirect relationship between laissez-faire leadership and subordinates’ performance *via* subordinates’ hindrance appraisal will be negative; and (2) when subordinates’ performance-prove or performance-avoid goal orientation is high, the relationship between laissez-faire leadership and challenge appraisal will be positive; the indirect relationship between laissez-faire leadership and subordinates’ performance *via* subordinates’ challenge appraisal will be positive. This study found the double-edged sword effect of laissez-faire leadership at within-person level, which helps integrate inconsistent views in previous studies and explore the impact of laissez-faire leadership from a more nuanced and balanced perspective.

## 1. Introduction

Leaders play a vital role in organizations. In order to maintain the efficient operation of the organization, leaders need to undertake important functions such as setting goals, motivating subordinates, participating in decision-making, and giving feedback (Bass and Bass, 2009). However, as leadership behavior is influenced by dynamic factors (such as cognition and affect), it is common to see leaders being rendered unable to lead in organizations now and then (Barnes et al., 2015).

Laissez-faire leadership reflects “non-leadership” state in organizations. According to Bass and Avolio (1995), laissez-faire leadership is described as “the absence of leadership, the avoidance of intervention, or both.” Laissez-faire leaders avoid making decisions, resist expressing opinions, hesitate about taking action and are absent when needed (Bass and Avolio, 1994; Judge and Piccolo, 2004; Hinkin and Schriesheim, 2008).

To date, laissez-faire leadership has primarily been considered as the most passive leadership style that may yield various destructive effects on employees (Bass and Avolio, 1997; Hinkin and Schriesheim, 2008; Skogstad et al., 2014; Hu et al., 2022; Parveen et al., 2022). However, several studies have suggested that laissez-faire leadership may not necessarily lead to negative outcomes. For instance, with regards to the relationship between laissez-faire leadership and motivation, Chaudhry and Javed (2012) indicated that laissez-faire leadership has a positive but not significant relationship with employee motivation, whereas Zareen et al. (2015) and Fiaz et al. (2017) both found that laissez-faire leadership has a significant positive impact on motivation. Furthermore, Pahi et al. (2018) found that laissez-faire leadership is beneficial to doctors’ commitment to service quality. In addition, Breevaart and Zacher (2019) illustrated that when leaders show both transformational and laissez-faire leadership, subordinates will have higher trust in leaders. Tong (2020) found that laissez-faire psychological leadership has a positive effect on organizational learning ability. Recently, Oprea et al. (2022) found out that laissez-faire leadership may lead to positive job crafting behaviors. Jamali et al. (2022) illustrated a positive impact of laissez-faire leadership on faculty performance in academic institutions. Md Rami et al. (2022) found that laissez-faire leadership is beneficial to social capital.

How might these inconsistent findings be explained? One of the possible explanations could be that there are boundary conditions under which laisse-faire leadership may have different effects on their subordinates. Drawing from the stress theory (Lazarus and Folkman, 1984) and achievement goal theory (Dweck, 1986), the double-edged sword effect of laissez-faire leadership was proposed in this study.

**Figure 1:**
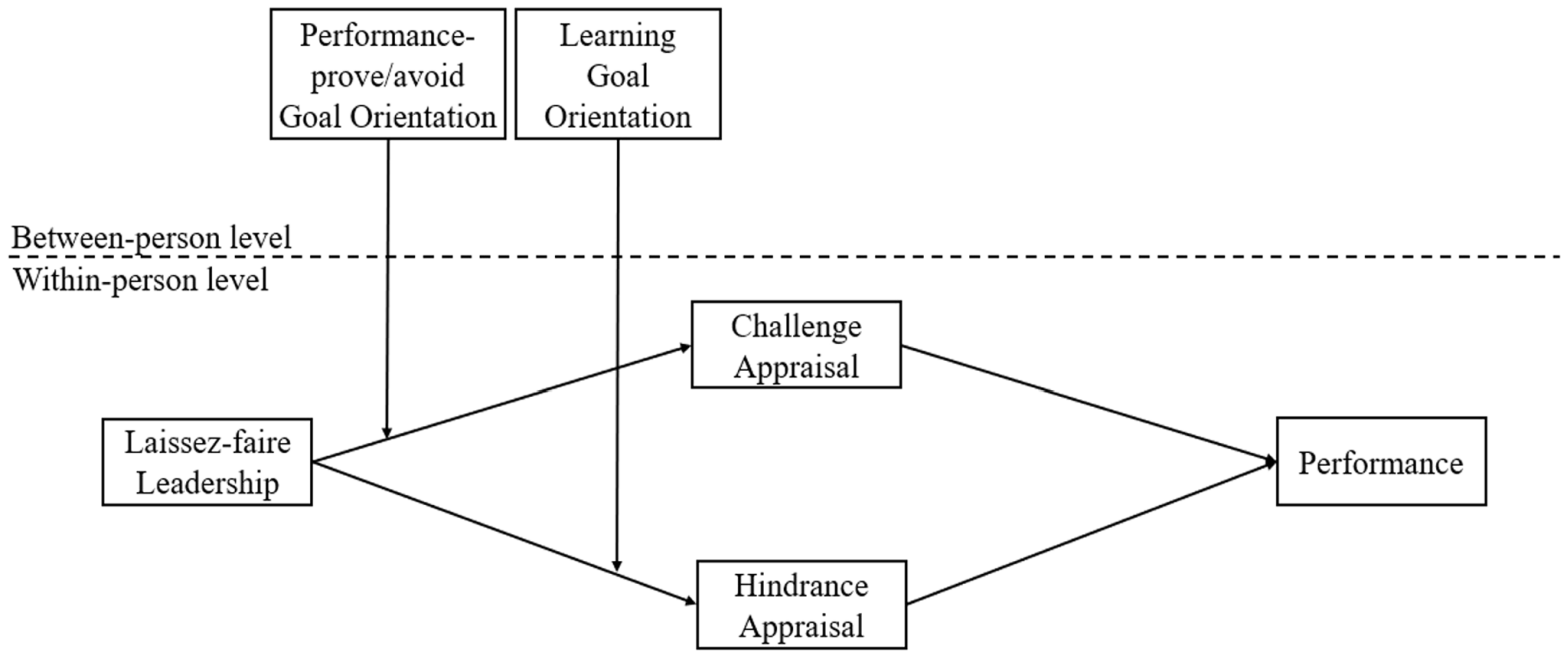
Hypothesized model.

According to the stress theory, individuals’ cognitive appraisals are determined by the extent to which an environmental event threats or facilitates their personal goals (Lazarus and Smith, 1988). Therefore, although laissez-faire leadership is predominately considered as one of the important workplace stressors (Skogstad et al., 2007), subordinates with different achievement goals (i.e., learning goal orientation and performance-prove/avoid goal orientation) may generate different cognitive appraisals (i.e., challenge appraisal and hindrance appraisal) in response to such stressor, which may lead to the change of job performance accordingly. Depicted in Figure 1 is the hypothesized model of this study.

Because people continuously appraise and cope with events in their day-to-day life (Lazarus and Folkman, 1984), their challenge/ hindrance appraisal of a singular stressor may vary over time. Some empirical studies have provided evidence for this point of view (e.g., Rosen et al., 2020). Therefore, to capture the daily fluctuations in subordinates’ cognitive state and performance outcomes, the experience sampling methodology (ESM; Bolger et al., 2003) was adopted in this study.

This study contributes to leadership research in three ways. Firstly, this research explores the double-edged sword effect of laissez-faire leadership and examines the boundary conditions, providing a more nuanced and balanced understanding of this phenomenon. Existing research neglected the fact that people differ in interpretations and reactions when faced with the same passive leadership. Specifically, laissez-faire leaders will lead to different appraisals of subordinates with different goal orientations, which will have indirect positive and negative effects on subordinates’ work performance. Thus, this study offers insights to explain why and how laissez-faire leadership may have both bright and dark side.

Secondly, in response to researchers’ call, this research explains how supervisors might influence subordinates’ stress in a cognitive way (LePine et al., 2016). As noted by LePine et al. (2016), it is well established that positive leader behaviors such as support and trust are associated with lower levels of subordinates’ stress, while negative leader behaviors such as abusive and laissez-faire leadership are associated with higher subordinates’ stress. However, a clear understanding of how leaders might influence subordinate appraisals of stressor and what consequences such appraisals will lead to is lacking. The current research highlights the critical role that cognitive appraisal plays in determining leadership outcomes by revealing the fact that laissez-faire leadership is conceptualized as a stressor though, subordinates with different goal orientation might appraise such leader behaviors in different ways, consequently increasing or decreasing their performance.

Finally, this research examines the fluctuations of laissez-faire leadership within one day. Previous studies mainly focused on the average level of laissez-faire leadership between different leaders (e.g., Judge and Piccolo, 2004; Robert and Vandenberghe, 2021). However, such perspective ignored the fact that the same leader may display varying degrees of laissez-faire leadership in different periods or even within one day. Adopting a within-person perspective, this study reveals the dynamic nature of laissez-faire leadership, capturing the immediate effect that daily laissez-faire leadership has on the subordinates, which helps improve the accuracy of laissez-faire leadership research (McCormick et al., 2020).

## 2. Theory and hypotheses

### 2.1. Laissez-faire leadership and subordinates’ challenge/hindrance appraisal

Laissez-faire leadership is conceptualized as the avoidance and/or absence of leadership (Avolio and Bass, 2001). With laissez-faire leadership, there are generally neither transactions nor agreements with followers. Their decisions are often delayed; feedback, rewards, and involvement are absent; and there is no attempt to motivate followers or to recognize and meet their needs (Skogstad et al., 2014). Therefore, laissez-faire leadership is generally considered as the most passive and ineffective form of leadership (Antonakis et al., 2003).

Resulting from the avoidance and absence of leadership, laissez-faire leadership is proven to be positively associated with subordinates’ role conflict, role ambiguity, and conflicts with coworkers (Kelloway et al., 2005; Skogstad et al., 2007). Therefore, extant research has conceptualized laissez-faire leadership as a root cause of workplace stressor (e.g., Kelloway et al., 2005; Diebig et al., 2016; Lundmark et al., 2022). What remains unknown is whether there are conditions under which subordinates may have different appraisals for such stressor, and what consequences those appraisals will lead to.

According to the stress theory, Lazarus and colleagues highlighted that whether a stressful event is appraised negatively as threatening or positively as challenging is contingent on what the person wants, that is, whether it thwarts or facilitates his/her personal goals (Lazarus and Folkman, 1984; Lazarus and Smith, 1988). Extending Lazarus and Folkman’s (1984) theory, LePine et al. (2016) identified two types of cognitive appraisals, namely the challenge appraisal and hindrance appraisal, to reflect employees’ subjective interpretation of workplace stressor. Specifically, challenge appraisals refer to an individual’s subjective perception of the work stressor that has a potential for personal gain, growth, development, and well-being, whereas hindrance appraisals refer to an individual’s subjective perception of the work stressor that has a potential to result in personal loss, constraints, or harm (LePine et al., 2016).

Given that subordinates with different personal goals may perceive and appraise laissez-faire leadership in opposite ways, this study proposes that the relationship between supervisors’ laissez-faire leadership and subordinates’ cognitive appraisal (i.e., challenge and hindrance appraisal) is contingent on subordinates’ goal orientation.

### 2.2. The moderating role of subordinates’ goal orientation

According to achievement goal theory, goal orientation refers to the goals people pursue in achievement situations (Dweck, 1986; Vandewalle, 1997). As Dweck and Leggett (1988) noted, because the goals pursued by an individual create a framework for interpreting and responding to events that occur, the same event may have an entirely different meaning and impact if it happens to people with different goals. Plenty of studies have shown that goal orientation affects how people interpret and respond to their work environment (e.g., Ma et al., 2021; Noskeau et al., 2021; Chi and Lam, 2022; Zhang et al., 2022). Vandewalle (1997) studied employee goal orientation in workplace and outlined three subordinate goals in workplace, namely learning goal orientation (LGO), performance-prove goal orientation (PPGO), and performance-avoid goal orientation (PAGO). To date, organizational research has widely tested and validated the 3-factor conceptualization (e.g., Wang et al., 2018; Ma et al., 2021).

There is an ongoing debate on whether goal orientation is a trait or a state (Vandewalle et al., 2019). In the current research, this study focus on trait goal orientation, which is defined as one’s consistent pursuits across achievement situations (Vandewalle, 1997). This is because, according to the stress theory, it is individuals’ enduring dispositions that govern their appraisal process and determine their reactions to stressors (Lazarus and Folkman, 1984).

Below is the proposal of how laissez-faire leadership and goal orientation jointly influence subordinates’ cognitive appraisal.

#### 2.2.1. Learning goal orientation

LGO is a desire to enhance one’s ability, improve competence, and experience mastery in achievement situations (Vandewalle, 1997). Individuals with higher LGO focus on the goal of demonstrating incremental improvement of themselves. To achieve a learning goal, subordinates proactively seek feedbacks and information from others, because they believe feedbacks, especially negative or critical ones, help locate their disadvantages and teach them how to improve their performance and behavior (Vandewalle and Cummings, 1997; Vandewalle et al., 2000; Alexander and van Knippenberg, 2014; Miron-Spektor et al., 2022). Learning goal also motivates subordinates to focus on developing new skills, attempting to understand their tasks, and successfully achieving self-referenced standards for mastery (Vandewalle and Cummings, 1997; Ford et al., 1998). By doing so, subordinates can continue to make progress in the self-development.

Laissez-faire leadership hinders subordinates from achieving their learning goals. Specifically, laissez-faire supervisors avoid interacting with their subordinates (Bass and Avolio, 1990). Due to the lack of interpersonal communication, subordinates are unable to receive constructive feedbacks and learn from past experience. Laissez-faire supervisors also shrink their input on motivating or coaching their subordinates (Skogstad et al., 2014), which is detrimental to subordinates’ mastery of tasks and achievement of extraordinary goals. Additionally, laissez-faire supervisors do not set clear goals (Frischer, 2006), as opposed to the desire of learning-orientated subordinates who want to take on difficult tasks (Payne et al., 2007). Furthermore, role ambiguity and interpersonal conflict resulting from laissez-faire leadership might distract subordinates from focusing on developing new skills and understanding their tasks, leading to an unnecessary waste of time and energy. Recent empirical studies also found that learning-oriented employees may have negative experience when the job provides few opportunities to acquire new skills (Ju et al., 2021). Therefore, subordinates who have higher LGO are more likely to appraise laissez-faire leadership as hindrance against their personal goals than subordinates who have lower LGO.

*Hypothesis 1a*: laissez-faire leadership and subordinates’ LGO interact to predict subordinates’ hindrance appraisal, such that laissez-faire leadership is more positively linked to subordinates’ hindrance appraisal when subordinates’ LGO is high rather than low.

#### 2.2.2. Performance-prove goal orientation

Performance-prove goal orientation (PPGO) reflects a desire to demonstrate one’s competence, exhibit excellent performance, and gain favorable judgments from others (Vandewalle, 1997). Both LGO and PPGO are construed as approach goals, in that both focus on success (Ames, 1992; Elliot et al., 2017). Unlike LGO, PPGO is the desire to “look successful” rather than engaging in activities that could help them actually enhance abilities and skills (Vandewalle et al., 2001). In order to look successful, subordinates with strong PPGO prefer familiar tasks because such tasks are easier for them to perform well (Vandewalle et al., 2001). Moreover, subordinates with strong PPGO prefer solving problems independently, because they view seeking input (such as assistance and feedback) from others as a sign of low ability (Vandewalle and Cummings, 1997). PPGO also motivates subordinates to take charge as long as they believe that doing so can make themselves look good to others (Hirst et al., 2011).

Laissez-faire leadership serves as favorable conditions for subordinates to pursue PPGO. Firstly, laissez-faire leadership allows subordinates to have autonomy (Sorenson, 2000; Chaudhry and Javed, 2012; Yang, 2015; Pahi et al., 2018). Without supervisors’ high requirements and close monitoring, high performance-oriented subordinates can do familiar tasks in the way they prefer, making them look successful. Secondly, laissez-faire supervisors shrink their leadership duties and leave much responsibility to subordinates, enabling them to take over the influence of such supervisors (Bass and Bass, 2009). By making decisions or taking sides in disputes when their supervisor is absent, subordinates may demonstrate their leadership potential. For example, empirical evidence from decentralized organizations shows that prove-oriented people will construe team discussions as a forum in which they demonstrate competence (Hirst et al., 2011). The chance to display their prowess may tantalize them to display high levels of proficiency and to be acknowledged and recognized for their abilities (Hirst et al., 2011). Therefore, although laissez-faire leadership results in role ambiguity and interpersonal conflict, subordinates who hold strong PPGO may interpret such stressor as challenging work demands that are beneficial for their personal goals.

*Hypothesis 1b*: laissez-faire leadership and subordinates’ PPGO interact to predict subordinates’ challenge appraisal, such that laissez-faire leadership is more positively linked to subordinates’ challenge appraisal when subordinates’ PPGO is high rather than low.

#### 2.2.3. Performance-avoid goal orientation

Performance-avoid goal orientation (PAGO) reflects a desire to conceal one’s incompetency, performance failure, and reduce negative evaluations (Vandewalle, 1997). Just like the two sides of a coin, the desire to “look successful” (i.e., PPGO) and the fear of “looking bad” (i.e., PAGO) are two sub-dimensions of a performance goal (Vandewalle, 1997). Empirical researchers have found that the effects of PPGO and PAGO on individuals’ perception in achievement situations tend to be similar (Payne et al., 2007). For example, both PPGO and PAGO make subordinates consider feedback from managers as unfavorable, and consider feedback-seeking behavior as an indicator of low ability (Vandewalle, 2003; Vandewalle et al., 2019). What differentiates PAGO from PPGO, however, is that subordinates who hold strong PAGO strive to avoid engaging in challenging tasks for fear of being seen as a failure by others (Payne et al., 2007).

Laissez-faire leadership may create the most comfortable environment for subordinates who hold strong PAGO. With laissez-faire leadership, subordinates who hold strong PAGO may be pleased to find that the supervisor he/she strives to avoid is avoiding him/her too. Because laissez-faire supervisors are indifferent to subordinates’ performance (Bass and Bass, 2009), such lack of supervision and evaluation may largely reduce subordinates’ anxiety about exposing incompetence to their supervisors. Moreover, laissez-faire supervisors do not set clear goals (Frischer, 2006; Skogstad et al., 2007), so subordinates are not required to achieve extraordinary performance, which further reduces their fear of failing to meet their supervisors’ expectations.

*Hypothesis 1c*: laissez-faire leadership and subordinates’ PAGO interact to predict subordinates’ challenge appraisal, such that laissez-faire leadership is more positively linked to subordinates’ challenge appraisal when subordinates’ PAGO is high rather than low.

### 2.3. The moderated indirect effect of laissez-faire leadership on performance

Challenging appraisal has a positive impact on work motivation, which in turn, improves work performance (LePine et al., 2005; Podsakoff et al., 2007; Li et al., 2018; Yan et al., 2022). Specifically, on the one hand, subordinates who generate challenge appraisal have positive expectations of the effects of their efforts, and believe that the desired results can be achieved by devoting time and energy (LePine et al., 2005; Wallace et al., 2009). On the other hand, those subordinates who also have positive expectations of the value of work returns believe that the time and effort they put in will help them achieve their goals (Podsakoff et al., 2007; LePine et al., 2016). When an individual believes that the work task is within his/her capabilities and the results are conducive to his/her development and growth, such positive expectation will motivate the individual to be more dedicated and active in their work, and they will strive to improve work performance (Prem et al., 2017; Liu and Li, 2018).

On the contrary, hindrance appraisal will inhibit the subordinates’ work motivation and negatively affect their performance (Rosen et al., 2020; Ma et al., 2021; Wang et al., 2022). When subordinates generate hindrance appraisal, they do not believe that efforts can achieve desired results, and believe that the difficulties and obstacles at work are insurmountable, thus losing confidence in work, leading to distraction and easy abandonment (LePine et al., 2005; Wallace et al., 2009). At the same time, hindrance appraisal also makes individuals deny the value of work rewards, believing that hard work is not helpful to their growth and development, thus losing enthusiasm for work, and leading to reduced time and energy (Liu and Li, 2018). In addition, studies have shown that hindrance appraisal may also lead to the exhaustion of individual cognitive and emotional resources, causing the individual to fall into a state of fatigue, anxiety and emotion exhaustion, and the energy needed to complete the work cannot be mobilized (Prem et al., 2017; Charkhabi, 2019). Under the combined influence of those liabilities, the individual’s work motivation is insufficient, which in turn leads to a decline in work performance.

Based on the analysis above, personal goals (i.e., goal orientation) will affect subordinates’ perception and evaluation (i.e., challenge appraisal or hindrance appraisal) of environmental events (i.e., laissez-faire leadership), thereby affecting their behavioral reactions (i.e., subordinates’ performance).

*Hypothesis 2a*: laissez-faire leadership and subordinates’ LGO jointly and indirectly predict subordinates’ performance via subordinates’ hindrance appraisal, such that the indirect effect is more negative when subordinates’ LGO is high rather than low.

*Hypothesis 2b*: laissez-faire leadership and subordinates’ PPGO jointly and indirectly predict subordinates’ performance via subordinates’ challenge appraisal, such that the indirect effect is more positive when subordinates’ PPGO is high rather than low.

*Hypothesis 2c*: laissez-faire leadership and subordinates’ PAGO jointly and indirectly predict subordinates’ performance via subordinates’ challenge appraisal, such that the indirect effect is more positive when subordinates’ PAGO is high rather than low.

## 3. Methods

### 3.1. Sample and procedures

Experience sampling methodology (ESM) was used in this study. With the design of ESM, the within-person effects of laissez-faire leadership on subordinates’ challenge/hindrance appraisals and performance could be examined. In addition, subordinates’ goal orientation, as a relatively stable personal trait, could be tested for its cross-level moderating effect.

The sample of this study comes from 10 state-owned enterprises located in China. The survey was completed by 73 dyads of supervisors and their direct subordinates, who provide a total of 616 daily observations. Each daily observation includes matching data completed by supervisors and their subordinates.

Following the standard procedure of ESM, data collection was conducted in two phases. First, subordinates were asked to complete a one-time baseline survey that assessed their goal orientation. Second, 1 week following the baseline survey, participants completed daily online surveys (sent via e-mail) over a period of 2 weeks (10 consecutive days, Monday to Friday). The online survey link was sent to each participant via email every day. Specifically, at 5:00 p.m. each afternoon, subordinates were asked to report challenge and hindrance appraisals, and supervisors to report laissez-faire leadership and subordinates’ performance. To ensure the accuracy of measurements, daily online surveys were only accessible before 12 a.m. each day and participants were informed of the deadline.

Data screening and cleaning was conducted in following steps. Firstly, questionnaires completed by participants at the wrong time were deleted from the dataset. For example, the participants may have missed the survey on the first day and filled in the previously missed survey next day. Secondly, questionnaires that took too long or too short to fill in were dropped from the dataset; Thirdly, the validity of the data was assessed to determine if there were any instances of identical responses. After these steps, five supervisor-subordinate dyads provided less than two consecutive surveys and were thus removed from the data, leaving 68 supervisor-subordinate dyads in the final sample. Of the 68 supervisors, 78.0% were male, whose average age was 42.6 years (SD = 6.95). Of the 68 subordinates, 53.0% were male, whose average age was 33.9 years (SD = 7.78). The final sample of this study comprised of 616 paired usable observations, yielding a 95.1% overall response rate (of 680 possible paired responses).

### 3.2. Measures

The scale was translated and back translated according to Brislin’s (1980) procedure. All measures were rated on a 5-point Likert scale ranging from 1 (strongly disagree) to 5 (strongly agree).

Goal Orientation: Subordinates’ goal orientation was self-reported by subordinates using the 13-item measures developed by Vandewalle (1997), which include learning goal orientation (5 items, α = 0.91), performance-prove goal orientation (4 items, α = 0.81) and performance-avoid goal orientation (4 items, α = 0.70). Sample: “I often look for opportunities to develop new skills and learn new knowledge” (LGO), “I like to engage in projects that can prove my work ability to others” (PPGO), and “when I undertake a task that may expose my ability, I will be very worried” (PAGO).

Laissez-faire Leadership: Laissez-faire leadership was self-reported by supervisors using Bass and Avolio (1997) 4-item measure (α = 0.90). A sample item was “Today I avoid getting involved when important issues arise.”

Challenge/hindrance Appraisal: Subordinates’ daily challenge/hindrance appraisal were assessed with two 3-item measures developed by LePine et al. (2016). Challenge appraisal sample: “Today, I feel the demands of my job challenge me to achieve personal goals and accomplishment” (α = 0.94). Hindrance appraisal sample: “Today, I feel the demands of my job constrain my achievement of personal goals and development” (α = 0.96).

Performance: Subordinates’ daily performance was assessed by leader with a single item. Sample: “Today this subordinate performed his/her job well.” This item has been used by previous researchers (Bakker and Xanthopoulou, 2009; Yang et al., 2016).

## 4. Results

### 4.1. Confirmatory factor analysis results

Before conducting multilevel confirmatory factor analyses (MCFAs), ICC for each variable was calculated. ICC1 was 0.35 for laissez-faire leadership, 0.49 for challenge appraisal, 0.41 for hindrance appraisal, and 0.44 for job performance. These results revealed that intraindividual fluctuations explained significant amount of variance of outcome variables, indicating that multilevel modeling approach is appropriate for this study.

To evaluate model fit for CFA, Comparative fit index (CFI), Tucker-Lewis index (TLI), Root-mean-square error of approximation (RMSEA), and Standardized root-mean-square residual (SRMR) were used. To demonstrate acceptable model fit, the values of TLI and CFI should be greater than 0.95, SRMR should be less than 0.08, and RMSEA should be less than 0.06 (Hu and Bentler, 1999). Akaike information criteria (AIC) and Bayes information criteria (BIC) were used to allow comparison of nonnested models with the same variables. Relatively smaller values of AIC and BIC indicated better fit.

In this study, Mplus 7.4 was adopted to examine the distinctiveness among constructs by conducting multilevel confirmatory factor analyses. The hypothesized seven-factor model showed good fit indices ($\frac{\chi^{2}}{df}$= 2.28, CFI = 0.98, TLI = 0.98, RMSEA = 0.04, SRMR_within_ = 0.04, SRMR_between_ = 0.11, AIC = 7681.52, BIC = 7966.41). If any two factors in the seven-factor model were combined into one, then the fitting index could not reach the level of the seven-factor model (3.53 ≤ $\frac{\chi^{2}}{df}$ ≤ 29.10), supporting discriminant validity of the variables. The AIC and BIC of seven-factor model were also smaller than those of any other comparative models, indicating that the proposed model fit the data better.

### 4.2. Descriptive statistics and correlations

Means, standard deviations, correlation coefficients and reliabilities of variables are shown in Table 1. As noted, the relationship between laissez faire leadership and challenging appraisal (*r* = −0.01, n.s.) and hindrance appraisal (*r* = 0.07, n.s.) is not significant, and challenge appraisal is positively related to performance (*r* = 0.18, *p* < 0.01), while hindrance appraisal is negatively related to performance (*r* = −0.21, *p* < 0.01). These results provide a preliminary basis for hypotheses testing.

**Table 1:** Descriptive statistics, correlations, and reliabilities.

Variable	Means	SD	1	2	3	4	5	6	7
Within-person variables	–	–	–	–	–		–	–	
1. Laissez-faire leadership	1.24	0.56	(0.90)	−0.01	0.07	0.04	–	–	
2. Challenge appraisal	3.84	0.78	−0.04	(0.94)	−0.15^**^	0.18^**^	–	–	
3. Hindrance appraisal	1.72	0.74	−0.01	−0.55^**^	(0.96)	−0.21^**^	–	–	
4. Performance	4.12	0.60	−0.05	0.52^**^	−0.31^**^	(−)	–	–	
Between-person variables	–	–	–	–	–	–	–	–	
5. Learning goal orientation	4.17	0.64	−0.02	0.37^**^	−0.21	0.07	(0.91)	–	
6. Performance-prove goal orientation	3.72	0.71	−0.00	0.24^**^	−0.14	−0.03	0.57^**^	(0.81)	
7. Performance-avoid goal orientation	2.84	0.66	−0.13	−0.10	−0.06	−0.15	−0.12	0.23	(0.70)

Within-person level, *N* = 616; Between-person Level, *N* = 68. Numbers above the diagonal are correlations at the within-person level. Numbers under the diagonal are correlations at the between-person level. Reliability coefficients are displayed on the diagonal in parentheses. For within-person variables, their reliabilities were the mean alphas across 10 days of observations.

**p < 0.01.

### 4.3. Hypothesis tests

Prior to the hypothesis testing, the sufficiency of within-person variances of laissez-faire leadership, challenge appraisal, hindrance appraisal and performance were examined. HLM7 was used to examine the proportion of within-person variance in total variance. Results suggested a substantial proportion of within-person variance in total variance: 63.39% for laissez-faire leadership, 50.48% for subordinates’ challenge appraisal, 58.22% for subordinates’ hindrance appraisal, and 52.24% for subordinates’ performance. Thus, it is reasonable to test these variables at within-person level and design cross-level moderating model.

In this study, Mplus 7.4 was used for multilevel path analysis. The results of H1a, H1b, and H1c are shown in Table 2.

**Table 2:** Results for the test of proposed model.

Variables	Challenge appraisal	Hindrance appraisal	Performance
Within-person level	–	–	–
laissez-faire leadership	0.10	0.04	0.06
challenge appraisal	–	–	0.12^**^
hindrance appraisal	–	–	−0.15^**^
Between-person level	–	–	–
learning goal orientation	0.29^*^	−0.18	0.08
performance-prove orientation	0.07	0.00	−0.01
performance-avoid orientation	−0.08	−0.08	−0.10
Cross-level interactions	–	–	–
laissez-faire leadership × learning goal orientation	−0.11	0.19^**^	–
laissez-faire leadership × performance-prove orientation	0.25^*^	−0.18^*^	–
laissez-faire leadership × performance-avoid orientation	0.19^*^	0.04	–

Within-person level, *N* = 616; Between-person Level, *N* = 68.

^*^p < 0.05. ^**^p < 0.01.

**Figure 2:**
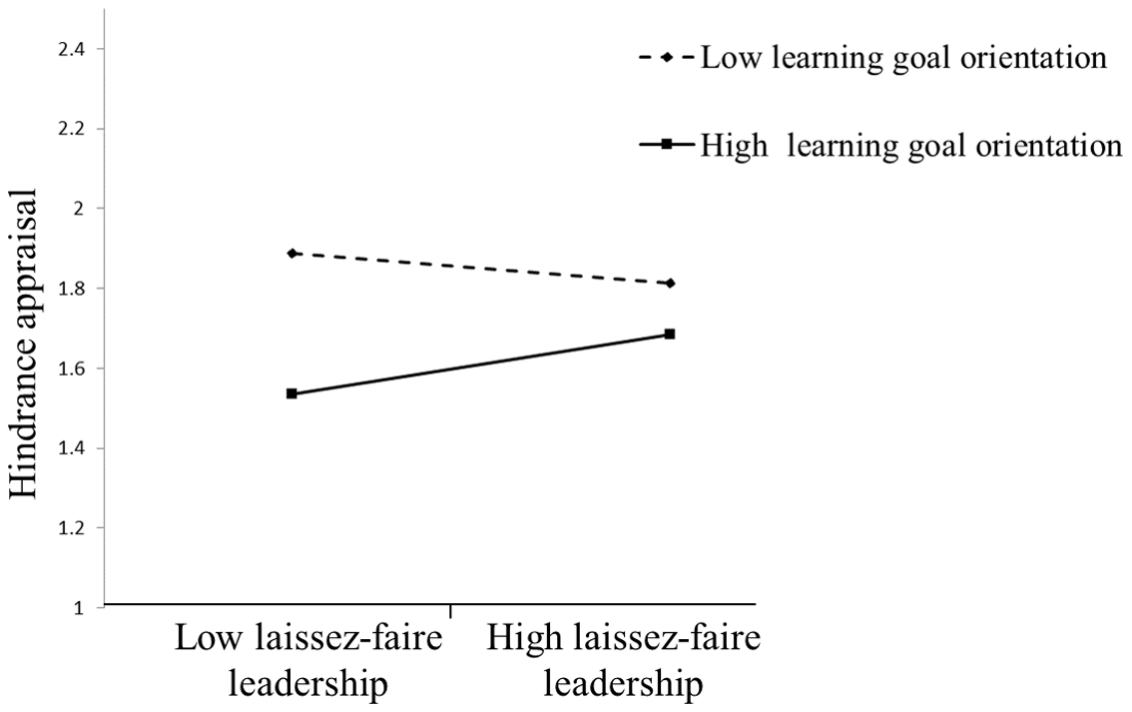
Learning goal orientation moderates the relationship between laissez-faire leadership and hindrance appraisal.

Hypothesis 1a predicted that subordinates’ learning goal orientation moderates the relationship between laissez-faire leadership and hindrance appraisal such that laissez-faire leadership is more positively related to hindrance appraisal when learning goal orientation is higher rather than lower. The results showed that the effect of laissez-faire leadership × learning goal orientation interaction term on hindrance appraisal was significant (*b* = 0.19, *p* < 0.01). Figure 2 shows that the direct effect of lasses-faire leadership on hindrance appraisal was positive when learning goal orientation was higher (*b* = 0.17, *p* < 0.05) rather than lower (*b* = −0.08, n.s.), supporting Hypothesis 1a.

**Figure 3:**
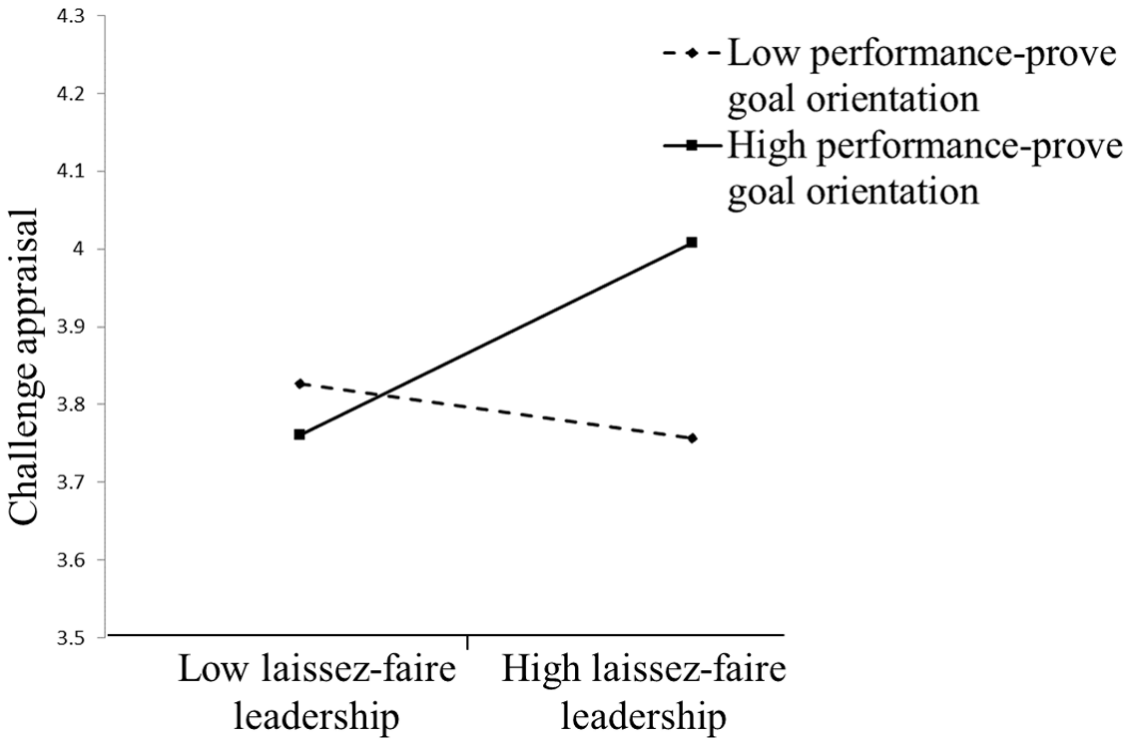
Performance-prove goal orientation moderates the relationship between laissez-faire leadership and challenge appraisal.

Hypothesis 1b predicted that subordinates’ performance-prove goal orientation moderates the relationship between laissez-faire leadership and challenge appraisal such that laissez-faire leadership is more positively related to challenge appraisal when performance-prove goal orientation is higher rather than lower. The results showed that the effect of laissez-faire leadership× performance-prove goal orientation interaction term on challenge appraisal was significant (*b* = 0.25, *p* < 0.05). Figure 3 shows the direct effect of laissez-faire leadership on challenge appraisal was positive when performance-prove goal orientation was higher (*b* = 0.28, *p* < 0.05) rather than lower (*b* = −0.08, n.s.), supporting Hypothesis 1b.

**Figure 4:**
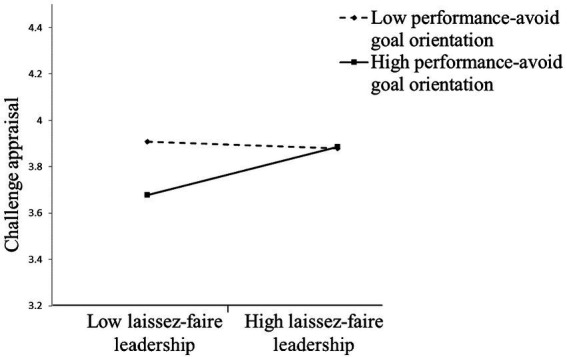
Performance-avoid goal orientation moderates the relationship between laissez-faire leadership and challenge appraisal.

Hypothesis 1c predicted that subordinates’ performance-avoid goal orientation moderates the relationship between laissez-faire leadership and challenge appraisal such that laissez-faire leadership is more positively related to challenge appraisal when performance-avoid goal orientation is higher rather than lower. The results showed that the effect of laissez-faire leadership× performance-avoid goal orientation interaction term on challenge appraisal was significant (*b* = 0.19, *p* < 0.05). Figure 4 shows the direct effect of laissez-faire leadership on challenge appraisal was positive when performance-avoid goal orientation was higher (*b* = 0.23, *p* < 0.05) rather than lower (*b* = −0.02, n.s.), supporting Hypothesis 1c.

To test Hypothesis 2a, Hypothesis 2b, and Hypothesis 2c, Monte Carlo Method was used to test the moderated mediation effect. Results are shown in Table 3.

**Table 3:** Mediated moderation results for laissez-faire leadership across levels of learning goal orientation and performance goal orientation.

Outcomes	Moderator	Mediator: Challenge appraisal			
			Conditional indirect effect	95% CI (Upper)	95% CI (Lower)
Performance	Learning goal orientation	High	0.00	−0.02	0.02
		Low	0.02	−0.00	0.05
	Performance-prove goal orientation	High	0.03^*^	0.00	0.07
		Low	−0.01	−0.03	0.01
	Performance-avoid goal orientation	High	0.03^*^	0.00	0.06
		Low	−0.00	−0.02	0.02
Outcomes	Moderator	Mediator: Hindrance appraisal			
			Conditional indirect effect	95% CI (Upper)	95% CI (Lower)
Performance	Learning goal orientation	High	−0.02^*^	−0.05	−0.00
		Low	0.01	−0.01	0.04
	Performance-prove goal orientation	High	0.01	−0.01	0.04
		Low	−0.03^*^	−0.06	−0.00
	Performance-avoid goal orientation	High	−0.01	−0.04	0.02
		Low	−0.00	−0.02	0.02

Within-person level, *N* = 616; Between-person Level, *N* = 68.

^*^p < 0.05. ^**^p < 0.01.

Hypothesis 2a proposed that learning goal orientation moderates the indirect effect of laissez-faire leadership on performance. When learning goal orientation is high, laissez-faire leadership has a more negative effect on performance through hindrance appraisal. The results showed that the learning goal orientation has a significant moderating effect on the indirect impact of laissez-faire leaders on subordinates’ performance through hindrance appraisal (Δ indirect effect = −0.03, 95%CI [−0.07, −0.01]). The indirect effect of laissez-faire leadership on subordinates’ performance via hindrance appraisal was positive when learning goal orientation was high (indirect effect = −0.02, 95%CI [−0.05, −0.00]), but this indirect effect was not significant when learning goal orientation was low (indirect effect = 0.01, 95%CI [−0.01, 0.04]), hence supporting hypothesis 2a.

Hypothesis 2b proposed that performance-prove goal orientation moderates the indirect effect of laissez-faire leadership on performance. When performance-prove goal orientation is high, laissez-faire leadership has a more positive effect on performance through challenge appraisal. The results showed that the performance-prove goal orientation has a significant moderating effect on the indirect impact of laissez-faire leaders on subordinates’ performance through challenge appraisal (Δ indirect effect = 0.03, 95%CI [0.01, 0.09]). The indirect effect of laissez-faire leadership on subordinates’ performance via challenge appraisal was positive when performance-prove goal orientation is high (indirect effect = 0.03, 95%CI [0.00, 0.07]), but this indirect effect was not significant when performance-prove goal orientation was low (indirect effect = −0.01, 95%CI [−0.03, 0.01]), hence supporting hypothesis 2b.

Hypothesis 2c proposed that performance-avoid goal orientation moderates the indirect effect of laissez-faire leadership on performance. When performance-avoid goal orientation is high, laissez-faire leadership has a more positive effect on performance through challenge appraisal. The results show that the performance-avoid goal orientation has a significant moderating effect on the indirect impact of laissez-faire leaders on subordinates’ performance through challenge appraisal (Δ indirect effect = 0.02, 95%CI [0.00, 0.07]). The indirect effect of laissez-faire leadership on subordinates’ performance via challenge appraisal was positive when performance-prove goal orientation was high (indirect effect = 0.03, 95%CI [0.00, 0.06]), but this indirect effect was not significant when performance-prove goal orientation was low (indirect effect = −0.00, 95%CI [−0.02, 0.02]), hence supporting hypothesis 2c.

## 5. Discussion

The main purpose of this study is to find the double-edged sword effect of laissez-faire leadership and challenge the prevailing view that laissez-faire leadership is always negative. This study proposed that subordinates’ different goal orientation will influence their cognitive appraisal of laissez-faire leadership, and subsequently having positive and negative effect on their performance. Experience sampling method was used to test theoretical model of this study and all hypotheses were supported. This study found that for subordinates with high learning goal orientation, laissez-faire leadership is harmful, which will lead to poor performance via hindrance appraisal. Interestingly, the results also indicated that for subordinates with high performance goal orientation, laissez-faire leadership is beneficial to their performance via challenge appraisal.

### 5.1. Theoretical contributions

This study has several theoretical contributions. Firstly, it contributes to the laissez-faire leadership literature, which has produced inconsistent findings on the outcomes of such leadership. The studies on laissez-fair leadership to date have demonstrated its destructive consequences on subordinates’ performance, attitudes and behavior (e.g., Judge and Piccolo, 2004; Skogstad et al., 2007; Diebig and Bormann, 2020; Hu et al., 2022), while several recent studies illustrated positive effects of laissez-faire leadership. For instance, Zareen et al. (2015) and Fiaz et al. (2017) both found that laissez-faire leadership has a positive effect on employee motivation. Recently, Oprea et al. (2022) found out that laissez-faire leadership is beneficial to job crafting. The limited examination of positive or negative impacts within one study is insufficient to comprehensively reflect the influence of laissez-faire leadership and fails to synthesize inconsistent perspectives. Therefore, it is crucial to examine the paradoxical effects of laissez-faire leadership within one study. The current study suggested that the paradoxical outcomes produced by laissez-faire leadership demonstrate its image as a double-edged sword. By adopting stress theory (Lazarus and Folkman, 1984) and achievement goal theory, this study sought to understand this paradox by exploring the role of subordinates’ goal orientation in the relationship between laissez-faire leadership and subordinates’ appraisals and performance. Specifically, laissez-faire leadership would be appraised as a hindrance or a challenge by subordinates with different goal orientations, which further positively or negatively contributes to subordinates’ performance. Thus, in light of stress theory (Lazarus and Folkman, 1984) and achievement goal theory, the study is helpful to understand the inconsistent results of laissez-faire leadership in existing research, offering a more comprehensive picture of its impact on employees.

Secondly, this study also extends research on laissez-faire leadership by addressing the mechanism underlying its impact on employees. Although the effects of laissez-faire leadership are relatively well-documented, the underlying mechanisms and the boundary conditions associated with such effects remain scarce (Robert and Vandenberghe, 2021). Several studies, which are exceptions, mainly focus on subordinates’ emotion, norms, role clarity, and LMX (McColl-Kennedy and Anderson, 2005; Skogstad et al., 2007; Robert and Vandenberghe, 2021; Hu et al., 2022; Lundmark et al., 2022) as mediators to offer explanations. For instance, Skogstad et al. (2007) illustrated that laissez-faire leadership may lead to role ambiguity and workplace conflict, further leading to workplace bullying. Hu et al. (2022) found that laissez-faire leadership is related to employee time theft through workplace time theft norms. These articles neglected to examine subordinates’ stress appraisals as the mechanism between laissez-faire leadership and work outcomes. However, since laissez-faire leadership in the workforce can be a source of stress for subordinates, the current study suggests that it is crucial to consider not only the stress itself but also subordinates’ personal appraisal of the stress when studying subordinates’ reactions toward laissez-faire leadership. Therefore, the current study enhances understanding of laissez-faire leadership’s effects by introducing a cognitive perspective, which points out the importance of subordinates’ cognitive appraisal (i.e., challenge or hindrance appraisal) in predicting the effects of laissez-faire leadership and whether the effects are detrimental is contingent on subordinates’ goal orientation.

Lastly, the dynamic approach to understanding laissez-faire leadership sheds light on its daily fluctuating nature. Whereas the majority of research, which adopted a between-person perspective, has considered laissez-faire leadership to be a consistent style, this study suggests that supervisors’ laissez-fair leadership vary from day to day (within-individual variance accounted for 63.39% of the total variance). This is in line with Bass and Bass (2009) who pointed out that many leaders will more or less present laissez-faire behaviors in work, implicating the fluid nature of laissez-faire leadership. In addition, within-individual studies are considered to be more accurate in capturing and reflecting the change as well as influence of leadership (Kelemen et al., 2020). Breevaart and Zacher (2019) found weekly fluctuations in laissez-faire leadership by weekly dairy study, which reflects the dynamic nature of laissez-faire leadership. To capture the fluctuation more precisely, we need to examine laissez-faire leadership at more precise time periods. Previous research has documented the dynamic nature of other passive leadership styles on daily basis, such as abusive supervision (Liao et al., 2021). Thus, this research advances the literature on laissez-faire leadership by adopting a daily perspective in understanding leader’s laissez-faire behaviors and their dynamic impacts on subordinates.

### 5.2. Practical implications

First of all, even though laissez-faire leadership is widespread in organizations and exhibits negative effects, the results of this study suggest that it could be a double-edged sword depending on subordinates’ goal orientation. The key lies in how subordinates perceive and evaluate laissez-faire leadership. On a daily basis, subordinates may evaluate it as beneficial (harmful) to their personal growth, generating a challenge (hindrance) appraisal. Therefore, in management practice, leaders should try to find ways to influence the cognitive evaluation of subordinates, guide their positive cognitive evaluation, and avoid the negative influence brought by laissez-faire leadership. Leaders may convey the positive message of laissez-faire behavior to employees, let them understand the benefits and opportunities of such behavior, and reduce the possibility of negative interpretation of laissez-faire behavior. Furthermore, leaders may also set up some informal leader positions in the organization to help them complete part of the coordination, supervision and guidance work, so as to further reduce the possible negative impact of laissez-faire leadership.

Secondly, this study has shown that goal orientation is an important boundary condition that affects leadership effectiveness and determines subordinates’ cognition and evaluation of laissez-faire leadership. Therefore, in management practice, the role of goal orientation should be attached with great importance. On the one hand, goal orientation is relatively stable, and it may not be dichotomized into absolute good or bad. Managers can assign different tasks to subordinates according to their goal orientations, so as to better stimulate their work motivation and performance. Specifically, for subordinates with high learning goal orientation, leaders may set goals and provide more feedback and guidance for them. Also, challenging tasks would be welcomed by those subordinates. However, for subordinates with high performance goal orientation, the feedback and guidance from leaders may be less preferred, and they should be assigned with more clear and specific work.

On the other hand, even though goal orientation is relatively stable, existing studies have also shown that it can be induced or changed by the environment or organizational context (Button et al., 1996). Therefore, leaders can intervene and guide the goal-oriented development of employees in a certain organizational context. Specifically, for employees who are engaged in work that are demanding and challenging, managers should emphasize the importance of learning goals, shifting subordinates’ focus to learning new skills and mastering the environment. In contrast, for subordinates who are assigned with relatively simple and fixed work that requires little intervention from the leaders, their performance goals and commitment to organizational goals should be emphasized.

### 5.3. Limitations and directions for future research

Despite the strengths of this research, there are several limitations in the study. Firstly, collecting data at the same phase may lead to common method variance bias. To test this problem, Harman’s single-factor test and ULMC (controlling for the effects of an unmeasured latent methods factor; Podsakoff et al., 2003) were used. According to Harman’s single-factor test, this paper conducted principal component analysis on all items of the within-person level and found that 35.28% of the variation can be explained by the greatest common factor, which is below the critical standard of 50%, indicating that the common method variance bias of the study is not of great concern. According to the method of UMLC, on the basis of the seven-factor model in this article, CFA with an added common method variance factor was conducted, and the correlation between group factors and the common method factor was set to zero. The model fit index has not been significantly improved. These results further proved that the common method bias problem of this paper does not constitute a concern. In addition, considering that this research focuses on leadership behavior, subordinates’ cognition, and work behavior within the same day, the design of measuring variables at the same time is relatively reasonable in order to better fit the research question. Subsequent research can further test whether the relationship among these variables changes over time. Secondly, the sample in this study are all from state-owned enterprises, thus suggesting that the external validity of the research findings is limited. Future research may collect samples from other types of companies or organizations to further test the findings of this research. Thirdly, due to the difficulty of collecting ESM data, the sample size is relatively small. Supervisors and their subordinates had to complete daily surveys over a period of 2 weeks (10 consecutive days, Monday to Friday). Actually, the sample size of this study is comparable to other ESM studies recently published in top-tier journals (e.g., Lanaj et al. (2021), within-person level = 645, between-personal level = 80; Simon et al. (2022), within-person level = 422, between-personal level = 53). Future studies could anticipate the required sample size by power analyses before conducting ESM studies.

Future research may also be expanded from the following aspects. Firstly, this research reveals that goal orientation serves as a watershed for the effectiveness of laissez-faire leadership, that is, subordinates may have completely different perceptions and responses to work in the face of laissez-faire leadership due to different goal orientation. Future research may examine the impact of goal orientation on the effectiveness of other leadership styles related to goals, such as directive leadership, participative leadership, supportive leadership, and achievement-oriented leadership, which are based on path-goal theory (House, 1996; Saleem et al., 2020). Secondly, this research provides empirical evidence for the relationship among laissez-faire leadership, challenge appraisal, and hindrance appraisal at the within-person level. Future research may further use experience sampling method to explore how laissez-faire leadership or other leadership styles influence the subordinates’ reaction at the within-person level. Finally, while laissez-faire leadership is certainly not desirable, the impact of laissez-faire leadership cannot be simply generalized, considering the complexity of the organizational environment, work content, and personal characteristics of subordinates. Future research may further explore other mechanisms and boundary conditions of laissez-faire leadership to gain a more objective and comprehensive understanding of the bright and dark sides of laissez-faire leadership.

## Data availability statement

The raw data supporting the conclusions of this article will be made available by the authors, without undue reservation.

## Ethics statement

Ethical review and approval was not required for the study on human participants in accordance with the local legislation and institutional requirements. Written informed consent for participation was not required for this study in accordance with the national legislation and the institutional requirements.

## Author contributions

JZ contributed to the conception and design of this study, and wrote the initial draft of the manuscript. YW contributed to data analysis and interpretation, including conducting statistical analyses and providing insights into the findings. FG provided important revisions to the manuscript, offering feedback on the organization and clarity of the paper. All authors contributed to the article and approved the submitted version.

## Funding

This study was supported by the National Natural Science Foundation of China (No. 72102228).

## Conflict of interest

The authors declare that the research was conducted in the absence of any commercial or financial relationships that could be construed as a potential conflict of interest.

## Publisher’s note

All claims expressed in this article are solely those of the authors and do not necessarily represent those of their affiliated organizations, or those of the publisher, the editors and the reviewers. Any product that may be evaluated in this article, or claim that may be made by its manufacturer, is not guaranteed or endorsed by the publisher.
